# Towards Personalized Sampling in Clear Cell Renal Cell Carcinomas

**DOI:** 10.3390/cancers14143381

**Published:** 2022-07-12

**Authors:** Claudia Manini, Estíbaliz López-Fernández, José I. López

**Affiliations:** 1 Department of Pathology, San Giovanni Bosco Hospital, 10154 Turin, Italy; claudia.manini@aslcittaditorino.it; 2 Department of Sciences of Public Health and Pediatrics, University of Turin, 10124 Turin, Italy; 3 FISABIO Foundation, 46020 Valencia, Spain; estibaliz.lopez@universidadeuropea.es; 4 Faculty of Health Sciences, European University of Valencia, 46023 Valencia, Spain; 5 Biocruces-Bizkaia Health Research Institute, 48903 Barakaldo, Spain

**Keywords:** multisite tumor sampling, intratumor heterogeneity, clear cell renal cell carcinoma

## Abstract

**Simple Summary:**

Intratumor heterogeneity (ITH) is a constant event in malignant tumors and the cause of most therapeutic failures in modern oncology. Since clear cell renal cell carcinoma (CCRCC) is a paradigm of ITH, an appropriate tumor sampling is mandatory to unveil its histological and genomic complexity. Several strategies have been developed for such a purpose, trading-off cost and benefit. Here, we propose an evolution of the previous multisite tumor sampling (MSTS) strategy based on the last findings in the spatial distribution of metastasizing clones. This new personalized MSTS pays special attention to sample by sectors peripheral zones of the tumor, where ITH is high.

**Abstract:**

Intratumor heterogeneity (ITH) is a constant evolutionary event in all malignant tumors, and clear cell renal cell carcinoma (CCRCC) is a paradigmatic example. ITH is responsible for most therapeutic failures in the era of precision oncology, so its precise detection remains a must in modern medicine. Unfortunately, classic sampling protocols do not resolve the problem as expected and several strategies have been being implemented in recent years to improve such detection. Basically, multisite tumor sampling (MSTS) and the homogenization of the residual tumor tissue are on display. A next step of the MSTS strategy considering the recently discovered patterns of ITH regionalization is presented here, the so-called personalized MSTS (pMSTS). This modification consists of paying more attention to sample the tumor periphery since it is this area with maximum levels of ITH.

## 1. Introduction

In these days of highly sophisticated medicine, simple things such as tumor sampling still matter. Pathologists are the specialists responsible for handling and sampling tumor specimens in such a way that crucial information of every tumor can be unveiled. A strategy adaptable to different patterns of tumor evolution, trading-off cost and benefit, is needed to maximize results and to respond to oncologists’ expectations [1]. Although tumor sampling is a key point applicable to every tumor type, this narrative focuses specifically on clear cell renal cell carcinoma (CCRCC) because of the previous experience of the authors in this area. In addition, CCRCC is a quite common neoplasm in daily practice and a well-known example of intratumor heterogeneity (ITH). The following paragraphs review the principal arguments supporting the necessity to update tumor sampling strategies and revisit possible alternatives for the progressive implementation of a so-called “precision sampling” [2]. 

CCRCC ranks in the top 10 list of the most frequent tumors in Western countries and remains a problem of major concern for many health systems. Roughly 79,000 new cases and 14,000 deaths are expected in USA in 2022 [3]. Traditionally chemo- and radio-resistant, only early detection and antiangiogenic and immune checkpoint blockade therapies, alone or in combination, have improved survival of CCRCC patients in the last decade. However, a significant proportion of these patients still die of disease, usually in the context of a metastatic disease. 

CCRCC is a paradigmatic example of ITH, which is the cause of most therapeutic failures to date. Genomic analyses have shown that CCRCC is a complex disease in which clonal and sub-clonal diversification is high across the tumor with many genetic alterations involving typically few regions. This fact was unveiled in the seminal paper published by Gerlinger et al. in 2012 [4], in which the authors performed exome sequencing, chromosome aberration analysis, and ploidy profiling in multi-regional samples of four patients with metastatic disease. Since then, a great many studies have brought to light the spatial and temporal dynamics governing the evolution of this tumor type and others. 

Although initially considered a purely stochastic process, tumor evolution in CCRCC seems to follow some deterministic pathways. In this sense, a recent analysis of 1206 regions of 101 cases has discovered up to seven evolutionary patterns correlated with patient prognosis [5]. *BAP-1* driven, multiple clonal drivers, and *VHL* wild-type tumors were shown to follow a punctuated evolutionary model with rapid progression and display high levels of chromosomal complexity and low levels of ITH. By contrast, the family of *PBRM1* mutated tumors showed a branched evolution with attenuated progression, with lesser chromosomal complexity and high ITH. An analysis of 575 primary and 335 metastatic regions in 100 CCRCC patients has shown that the metastatic ability of CCRCC is associated with 9p and 14q losses [6]. The same study has also shown that those neoplasms which show a punctuated evolution presented early, multiple metastases while those with a branching pattern develop late, solitary ones.

Punctuated and branching are terms referring to two different patterns of temporal evolution which come from the application of ecological principles to cancer. Under this perspective, a tumor is a huge community of different individuals including neoplastic cells and cells of the tumor microenvironment such as endothelia, tumor-associated fibroblasts, macrophages, tumor-associated lymphocytes, and others. These elements are permanently interacting one each other. At least four models of tumor evolution have been described so far: linear, branching, neutral, and punctuated [7]. Linear, branching, and punctuated are Darwinian-type models whereas neutral is considered non-Darwinian. Linear model refers to a step-wise temporal process in which all cancer cells progressively increase their malignancy. This pattern will generate tumors with very low ITH. In the branching type of evolution, tumor cells coming from the same ancestor temporarily acquire different mutations resulting in different clones which regionalize the tumor in different areas. This pattern will give rise to tumors with high ITH. The punctuated pattern of evolution, also called the “big bang” model, is the result of a genomic aberration generating a dominant clone with high fitness at the very early stages of tumor evolution. As a result, punctuated tumors are typically aggressive and show low levels of ITH. Finally, neutral evolution reflects an evolutionary pattern in which extreme clonal diversity (hyper-branching) develops resulting in tumors with very high ITH. 

ITH also impacts tumor microenvironment, including cancer associated fibroblasts, macrophages, and tumor infiltrating lymphocytes. For example, it has been demonstrated that the expression of PD-1, PD-L1, and other immune checkpoint markers may be highly variable across different tumor regions, a feature that can compromise the correct selection of patients for immune checkpoint blockade therapy if the tumor is not appropriately sampled [8]. An incomplete tumor sampling may lead to false negative results, thus ruling out patients for a beneficial therapy. In this sense, Khagi et al. observed non-expected good responses to anti-PD-L1 therapy in up to 17% of cases that apparently did not express PD-L1 in the immunohistochemical study [9] suggesting suboptimal analyses.

## 2. Classic Sampling Protocols

**Figure 1:**
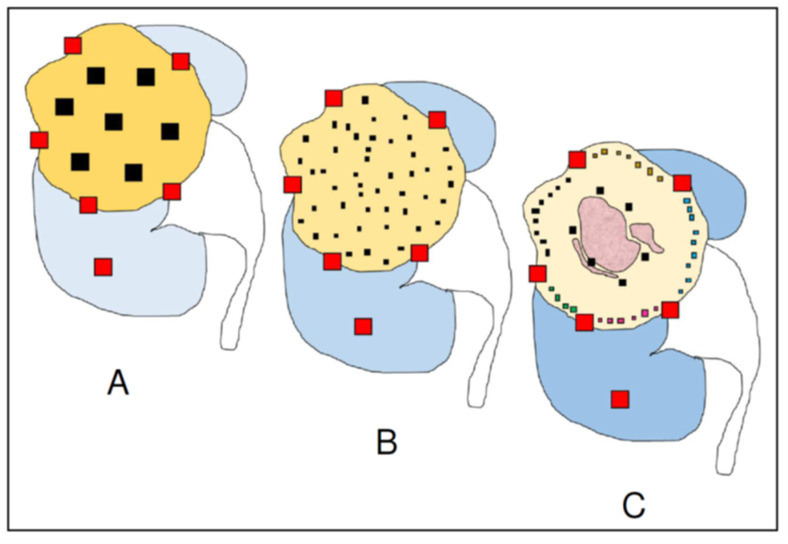
Schematic of tumor sampling evolution in clear cell renal cell carcinoma. The classical sampling protocol (**A**) calls for one block per centimeter of tumor diameter. Multisite tumor sampling (**B**) randomly selects a large number of small tumor samples across the tumor using the same number of blocks as the classic protocol. Advanced multisite tumor sampling (**C**) takes a few samples at the tumor center, where intratumor heterogeneity is low but metastatic genotypes and necrosis (pink areas) are common, and many small samples at the periphery, where intratumor heterogeneity and local invasiveness are high. Here, the small fragments selected are grouped in blocks by sectors, thus enabling the precise location of any key change in any sample to be monitored. Note that tumor/non-tumor, tumor/renal sinus, and tumor/perinephric fat interfaces are similar in the three methods (block shown in red).

Classic sampling protocols were designed decades ago when ITH detection was not a key issue for diagnosis and therapy. At that time, the recommendation was to obtain one tumor tissue sample per centimeter of tumor diameter plus samples from the tumor/non-tumor interface and from “suspicious” areas (Figure 1A) [10]. Those sampling protocols are not supported by any scientific observation and surprisingly survive nowadays in the era of precision medicine. 

Since ITH makes every tumor unique and unrepeatable, and next generation sequencing tools are demonstrating the real dimension of the genetic variability across a single tumor, the main question here should be: how much sampling is needed in every case? Total tumor sampling might be the perfect answer. This strategy may be affordable and advisable in small tumors (<3 cm), but it is not a realistic option in many tumors due to many of them are much larger. Some authors have suggested that sampling three distant regions would suffice to detect with a reliability of 90% of certainty key mutations in CCRCC such as those occurring in *PBRM1*, *SETD2*, *BAP1*, and *KDM5C* genes [11]. However, the number of samples should not be aprioristically fixed since it should vary with the size of the tumor. 

## 3. Multisite Tumor Sampling (MSTS)

**Figure 2:**
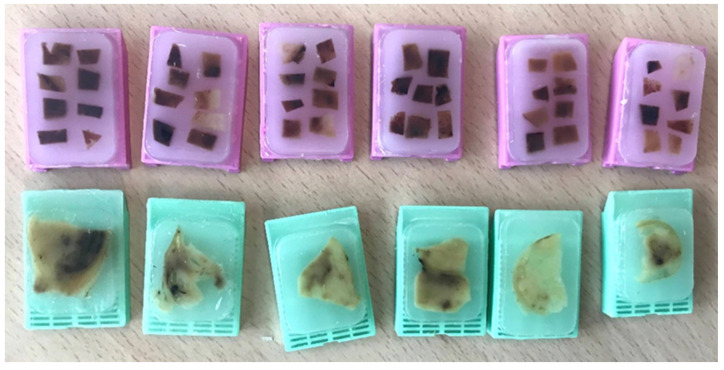
Multisite tumor sampling (pink blocks) consists on including six to eight small tumor tissue fragments in each paraffin block instead of one large tumor fragment proposed by the classic protocol (green blocks). This way, the same number of paraffin blocks sample many more tumor regions.

Classic sampling protocols seem insufficient in light of subsequent studies, which have suggested the convenience of a more thorough sampling to detect exome-wide driver events [5,6]. For this reason, a new, affordable strategy for trading-off cost and benefit was developed in 2016 [12]. It is called multisite tumor sampling (MSTS) (Figure 1B) and is based on the divide-and-conquer principle [13], a mathematical algorithm successfully used in such widely differing scientific fields as particle physics and medicine. The strategy consists in recursively breaking down a given problem into simpler parts (divide) until they are simple enough to be solved (conquer). Once the simple parts are solved they are all merged to resolve the initial problem. In our example, its application to tumor sampling consists of including six to eight small tissue fragments per block instead of a single large fragment (Figure 2). In this way, MSTS can afford to sample up to 48 tumor regions very distant from each other when sampling a 6 cm-in-diameter tumor, for example. In silico modelling comparing the performances and costs of the classic sampling protocol and MSTS confirms the superiority of the latter in detecting ITH at all temporal stages of tumor evolution [13,14]. A comparison of the performance in detecting histological features of bad prognosis such as high grade and granular eosinophilic cells [15] with both methods in 38 CCRCC showed that MSTS was significantly more informative than routine sampling [16]. 

Aside from CCRCC, the usefulness of MSTS in CCRCC has been confirmed by subsequent histological, immunohistochemical, and molecular studies in ovarian carcinoma, mesothelioma, and head and neck squamous cell carcinoma [17,18,19,20]. Lakis et al. [17] have analyzed 294 tumor sections from 70 treatment naïve patients who had undergone cytoreductive surgery of ovarian cancer and have observed not only the high histological variability of tumors across different regions, but also the irregular qualitative and quantitative distribution of tumor-associated lymphocyte, information with obvious prognostic and therapeutic implications. They conclude that ITH in ovarian cancer may limit the usefulness of pre-operative biopsies to make some therapeutic decisions. Meiller et al. [18] underline the usefulness of MSTS in detecting molecular ITH in malignant mesothelioma. MSTS performed in 16 patients from two different hospitals were analyzed both histologically, and by RT-PCR and targeted NGS. Mutational ITH, copy number variations and fusion transcripts, differential gene expression and signal pathway dysregulation, histo-molecular heterogeneity, epigenetic ITH, and tumor microenvironment were evaluated. The authors conclude that spatial ITH is high in malignant pleural mesothelioma and stress the convenience of analyzing different topographical areas of the tumor. This policy must be performed to better estimate the patient prognosis and the prediction of response to subsequent treatment. Jie et al. [19] have compared the performance of routine sampling and MSTS in 182 oral and oropharyngeal squamous-cell carcinomas. The authors included in the comparison histological, immunohistochemical, and molecular parameters, and concluded that MSTS was more informative than routine sampling in detecting perineurial permeation, peritumoral vascular/lymphatic growth, necrosis, muscle invasion, PIK3CA mutations (exons 9 and 20), and CDKN2A promoter methylation. Brunelli et al. [20] have recently compared a multi-regional sampling strategy called 3D fusion with routine sampling in 100 CCRCC analyzing the respective performance of both methods in the detection of angiogenic and immune markers. These authors confirm the superiority of 3D fusion sampling and agree that sampling one block/cm of tumor tissue diameter is inadequate to fully characterize ITH in CCRCC. Finally, another in silico study has shown the superiority of an adapted variant of the MSTS method in detecting tumor budding and intramural vasculo-lymphatic invasion in hollow viscera (urinary bladder and digestive) adenocarcinomas [21].

## 4. Homogenization of the Residual Tumor Tissue

Another attempt to improve genomic ITH detection in solid tumors has recently been made [22]. This method proposes the homogenization of the leftover residual tumor tissue before sequencing, thus guaranteeing the full genomic analysis of the whole tumor. However, this protocol has its limitations because not all surgical specimens generate enough representative leftover tissue after histological sampling. In addition, the topographic localization of the genomic data and its correlation with histology—a point that may be important—is lost after tissue homogenization. As it will be mentioned in the following paragraphs, leftover tissue homogenization will also negatively affect the precise topographic identification of the differences in the tumor microenvironment between tumor center and periphery, which are derived from differences in the hypoxic status, another crucial targetable point.

In a context of high diagnostic pressure, some pathologists may be reluctant to increase the time and cost needed to implement both 3D fusion [20] and the homogenization of the leftover residual tumor [22], a point that may limit their widespread implementation. By contrast, MSTS saves time because it is an *all-in-one* procedure, enabling at the same time the histological analysis with a genomic correlation to take place in the same paraffin block and preserving the formalin-fixed paraffin-embedded material for the future. Also, MSTS is an affordable method in public health systems since it does not increase the cost. If the paraffin block is considered the unit of cost in Pathology Labs, the MSTS’s cost is similar to the routine sampling because it employs the same number of blocks. For these reasons, MSTS is superior in terms of trading-off performance obtained and cost. 

## 5. Personalized Multisite Tumor Sampling (pMSTS) 

Recent findings on the spatial distribution of CCRCC clones and sub-clones [23,24] also suggest taking a step forward in searching for a more refined tumor sampling strategy. This evolution should look for constraints on cost and time, efficacy in the detection of histological and genomic data, and adaptability to adjust the procedure case by case. It should be noted that tumors are usually sampled without knowing the precise tumor landscape in every case. However, some broad findings in selected cases may supply useful data since predicting the possibility of aggressive forms of CCRCC may help in making sampling decisions. For example, spontaneous tumor necrosis is a common finding in large tumors which can be detected by the naked eye and is always related to high grade. Low ITH at histological and genomic levels are characteristic findings in many aggressive CCRCC [6,25]. In consequence, it can be inferred even in the grossing room that tumors showing areas of necrosis will have high-grade histological features and low levels of ITH.

A study of 756 mapped regions of 101 CCRCC has shown that the copy number alteration burden, percentage of necrosis, and histological grade are higher in the tumor interior, which is also where the metastasizing subclones preferentially develop, probably as a survival response to local environmental hypoxic pressures [23]. Moreover, a model enabling the development of clonal diversity in space and time of these tumors to be understood has been developed based on patterns of tumor growth and necrosis [24]. As a result, high ITH is located at the tumor periphery while the tumor interior remains relatively homogeneous. Since the conditions of hypoxia differ between the tumor center and periphery, tumor microenvironments adapt to the specific local necessities, displaying qualitative and quantitative variations in the innate and adaptive tumor immunities, tumor-associated fibroblasts, and other elements of this tumor compartment [26]. Interestingly, this model or center versus periphery distinction, at least in CCRCC, also has clinical implications since it connects radiological evidence of peripheral tumor budding in the early stages of tumor development with predictable future clonal evolution [24].

A more precise tumor sampling requires investing some extra time in the grossing room paying special attention to the macroscopic characteristics of the tumor, including size and shape, tumor margins, and allowing the detection of other tumor features like tissue consistency and color that usually give additional interesting information. Taking these classic recommendations on mind, together with the latest findings on spatial tumor evolution and regionalization as a whole, an advanced version of the MSTS protocol will provide a more closely adapted approach at the time of sampling CCRCC (Figure 1C). Given that metastasizing clones related to tumor necrosis, high grade, and low ITH are mostly located in the tumor interior, several samples placed in one block would suffice to provide a reliable representation of this tumor area including tumor and non-tumor cells. Also, grey/whitish tumor areas with stiffer consistency indicating sarcomatoid dedifferentiation will be seen by naked-eye and then included within the high-grade tumor blocks. By contrast, the peripheral rim of the tumor is characterized by high levels of ITH [23,24]; for this reason, many small tissue fragments such as those of the MSTS are needed to provide a complete snapshot of the tumor periphery. What is more, these small tissue samples can be included in the blocks distributed by sectors, so the topographic location of any specific molecular alteration associated to prognosis or treatment can be determined with precision in every case. 

## 6. Conclusions

Tumor sampling strategies do impact significantly on the development and success of truly precision oncological therapies. To hit the target and achieve widespread implementation, this strategy must be easily affordable on one hand and trade-off costs and benefits on the other; otherwise, its implementation in many Pathology Labs will be at risk. Two variants of the MSTS adaptable to the macroscopic findings observed in the grossing room and the alternative option of a complete homogenization of the leftover residual tumor tissue are currently available. To note, they appear to be complementary, non-exclusive according to this Perspective.

## Data Availability

Data sharing is not applicable to this article.
